# High Bias Gas Flows Increase Lung Injury in the Ventilated Preterm Lamb

**DOI:** 10.1371/journal.pone.0047044

**Published:** 2012-10-08

**Authors:** Katinka P. Bach, Carl A. Kuschel, Stuart B. Hooper, Jean Bertram, Sue McKnight, Shirley E. Peachey, Valerie A. Zahra, Sharon J. Flecknoe, Mark H. Oliver, Megan J. Wallace, Frank H. Bloomfield

**Affiliations:** 1 Liggins Institute, University of Auckland, Auckland, New Zealand; 2 Royal Women’s Hospital, Parkville, Victoria, Australia; 3 The Ritchie Centre, Monash Institute of Medical Research, Clayton, Victoria, Australia; 4 Newborn Services, Auckland City Hospital, Auckland, New Zealand; 5 National Research Centre for Growth and Development, Auckland, New Zealand; 6 Department of Paediatrics: Child and Youth Health, Faculty of Medical and Health Sciences, University of Auckland, Auckland, New Zealand; University of Tübingen, Germany

## Abstract

**Background:**

Mechanical ventilation of preterm babies increases survival but can also cause ventilator-induced lung injury (VILI), leading to the development of bronchopulmonary dysplasia (BPD). It is not known whether shear stress injury from gases flowing into the preterm lung during ventilation contributes to VILI.

**Methods:**

Preterm lambs of 131 days’ gestation (term = 147 d) were ventilated for 2 hours with a bias gas flow of 8 L/min (*n* = 13), 18 L/min (*n* = 12) or 28 L/min (*n* = 14). Physiological parameters were measured continuously and lung injury was assessed by measuring mRNA expression of early injury response genes and by histological analysis. Control lung tissue was collected from unventilated age-matched fetuses. Data were analysed by ANOVA with a Tukey post-hoc test when appropriate.

**Results:**

High bias gas flows resulted in higher ventilator pressures, shorter inflation times and decreased ventilator efficiency. The rate of rise of inspiratory gas flow was greatest, and pulmonary mRNA levels of the injury markers, *EGR1* and *CTGF*, were highest in lambs ventilated with bias gas flows of 18 L/min. High bias gas flows resulted in increased cellular proliferation and abnormal deposition of elastin, collagen and myofibroblasts in the lung.

**Conclusions:**

High ventilator bias gas flows resulted in increased lung injury, with up-regulation of acute early response genes and increased histological lung injury. Bias gas flows may, therefore, contribute to VILI and BPD.

## Introduction

Preterm birth is the greatest cause of morbidity and mortality in newborn infants and rates of preterm birth are increasing worldwide. Up to 70% of babies born before 26 weeks’ gestation develop bronchopulmonary dysplasia (BPD), a debilitating lung disease that results in abnormal lung development and which is usually defined as an ongoing requirement for supplemental oxygen at 36 weeks’ postmenstrual age [1], [2]. Histologically, BPD is characterized by poor alveolarisation [3], [4], abnormal elastin deposition [3], [4], [5], fibrosis [4], [6], mesenchymal cell hyperplasia [4], [7] and abnormal capillary growth [4]. Although the causes of BPD are unknown, the majority of infants with BPD received mechanical ventilation, which is considered to be a major contributing factor through the induction of ventilator-induced lung injury (VILI) to the immature lung [2].

Mechanisms underlying VILI are thought to include barotrauma [8], [9], volutrauma [10], [11], atelectotrauma [9] and biotrauma [12]. Although this knowledge has led to advances in perinatal respiratory care [13], [14], [15], the incidence of BPD following extremely preterm birth has not decreased [2].

The potential role of rheotrauma in VILI, related to high gas flows in the delicate preterm airways, has not yet been investigated. Mechanical conventional ventilation involves forcible injection of gas into the lungs, achieved in most neonatal ventilators by a continuous or variable bias gas flow within the ventilator circuit. During inspiration, the expiratory valve closes and pressure builds in the ventilator circuit providing a pressure gradient for gas to flow into the lung. Neonatal ventilators applying a continuous bias gas flow are commonly set to flows of 6–12 L/min, but there is little evidence to support the use of these flows [16]. Furthermore, it is often assumed that bias gas flow is directly related to inspiratory flow in the pulmonary airways. However, many factors influence inspiratory flow, including the type of airflow (laminar vs turbulent), the pressure gradient between the ventilation circuit and the airways as well as the resistance and compliance of the respiratory system.We have previously demonstrated that bias gas flow in term healthy lambs is inversely related to inflation time and that optimal ventilator efficiency was obtained at bias gas flows much lower than currently used in many neonatal intensive care units [17]. It is not known, however, whether bias gas flows also affect lung injury or pulmonary dynamics in the very immature and poorly compliant lung. We report here that high bias gas flows affect cardiorespiratory parameters, decrease ventilation efficiency, result in histological changes consistent with lung injury and upregulate mRNA levels of the early injury response genes *EGR1*, *CYR61* and *CTGF* which are known to play a pivotal role in adult human lung injury [18], [19], [20] and which reliably indicate VILI in preterm lambs [21].

## Materials and Methods

### Experimental Protocol

#### *In vitro* study

The inter-relationships between gas flow into an artificial lung and (1) bias gas flow in the ventilator circuit, (2) respiratory system compliance and (3) respiratory system resistance, were examined using a mechanical ventilator (Babylog8000*plus,* Dräger Medical, Lübeck, Germany). Three test lungs of differing compliances (0.4, 0.8 and 1.3 mL/cmH_2_O) and four different airway resistances (no endotracheal (ET) tube and ET tube sizes of 2.5, 3.0 and 4.0) were used. Peak inspiratory pressure (PIP) was set at 35 cm H_2_O. Airway pressure and gas flow into the test lungs were measured directly from the ventilator and recorded continuously at 4 different bias gas flows (4, 8, 18 and 28 L/min).

#### Animal study

*Ethics statement:* Experiments were approved by the University of Auckland Animal Ethics Committee, approval number AEC03/2006/R450. Ewes received betamethasone (Schering-Plough, Wellington, New Zealand, 11.4 mg intramuscularly) 48 and 24 h prior to delivery of lambs via hysterotomy at 131–133 days’ gestation (term = 147 days). Lambs (weight 4.8±0.1 Kg) were intubated with a size 4.0 cuffed endotracheal tube (Mallinckrodt, Athlone, Ireland) and ventilation was commenced with randomly assigned bias gas flows of 8 (*n* = 13), 18 (*n* = 12) or 28 L/min (*n* = 14). Umbilical arterial and venous catheters were placed and anesthesia induced with alfaxalone (JUROX Pty. Ltd, Rutherford, Australia, 4 mg/Kg) and maintained with midazolam (Roche, Auckland, New Zealand, 0.4 mg/Kg) and ketamine (Parnell Laboratories, Auckland, New Zealand, 4 mg/Kg). Muscle relaxation was maintained with pancuronium (AstraZeneca, North Ryde, Australia, 0.1 mg/Kg, intravenous).

Lambs were ventilated for 2 h (Babylog8000*plus,* Dräger Medical, Lübeck, Germany) with heated (37 °C) and humidified gas in pressure support ventilation + volume guarantee (PSV+VG) mode. In this mode, inflation is terminated when the inspiratory flow falls to <15% of the peak inspiratory flow, indicating that the lungs are nearly fully inflated. Settings were: tidal volume 7 mL/Kg; positive end expiratory pressure 6 cmH_2_O; maximum positive inspiratory pressure (PIP) 50 cmH_2_O; maximum inflation time (Ti) 0.8 s, and frequency 40 breaths/min (increased if PaCO_2_>60 mmHg or pH <7.25). Supplemental oxygen was administered if SaO_2_ was <90%. Pneumothoraces were promptly drained percutaneously.

Inspiratory and expiratory flow (MLT300L pneumotachograph; ADinstruments, Dunedin, New Zealand), blood pressure, oxygen saturation and ventilator parameters (Ventview, Dräger Medical) were measured and recorded continuously. Ventilator efficiency index (VEI) was calculated as 3800/[(PIP-PEEP)×ventilator rate×PaCO_2_] [17] and oxygenation index (OI) as mean airway pressure (cmH_2_O)×FiO_2_ (%)]/PaO_2_ (mmHg). Arterial blood gases were measured every 15 minutes. Full blood counts were performed at 0, 30, 60 and 120 min after commencing ventilation. Data from lambs that died during the experimental period were excluded from these analyses (numbers analysed: 8 L/min, *n* = 11; 18 L/min, *n* = 11; 28 L/min, *n* = 14).

At 2 hours lambs were euthanized with pentobarbitone (Provet NZ Pty Ltd, Auckland, New Zealand, 90 mg/Kg). Additional non-ventilated lambs (n = 8) were euthanized prior to delivery to act as unventilated controls. Lung tissue was collected as described previously [7].

### Tissue Analyses

mRNA levels of early growth response factor 1 (*EGR1*), cysteine-rich 61 (*CYR61*) and connective tissue growth factor (*CTGF*) in lung tissue were measured by quantitative real-time PCR as described previously, utilising the delta-delta C_T_ method normalised to 18S rRNA as a housekeeping gene and expressed relative to mean levels of the gene of interest in control tissue [21]. PCR data were available for all 8 control lambs and all experimental lambs for which physiological data were analysed (8 L/min, *n* = 11; 18 L/min, *n* = 11; 28 L/min, *n* = 14).

Lung tissue was prepared for histological and immunohistochemical analysis as described previously [7]. Five lambs were randomly selected from each experimental group for histological analysis; all 8 control lambs were analysed. Elastin was identified using Hart’s resorcin-fuchsin stain (12) and collagen with Gordon and Sweet’s reticulum stain. Myofibroblasts, which play a role in connective tissue fibre deposition and secondary septal crest formation, were identified by immunohistochemical staining of alpha smooth muscle actin [22], [23]. Proportion of tissue stained positive for elastin, collagen and alpha smooth muscle actin, percentage of lung occupied by tissue and secondary septal crest density were calculated in 5 random images per section as described previously [7] by a single observer (KPB) blinded to the experimental group. Proliferating cells, staining positive for Ki67 [7], were counted on 10 images per section and expressed as a proportion of the total number of cells to determine a labelling index.

### Data Analysis

Data that were not normally distributed (EGR1 and CTGF mRNA levels) were transformed by taking the cube root to approximate a normal distribution prior to analysis with parametric techniques. Data are represented as mean ± SEM. For the *in vitro* study, a non-parametric multivariate analysis was used to analyse the effects of bias gas flow, airway resistance and respiratory system compliance on gas flow into the artificial lung. Physiological data from the animal study were analysed in 10 minute epochs, commencing 5 minutes before each blood gas measurement, and were analysed by ANOVA, including analysis of effect of time. Histological data were analysed by nested ANOVA and mRNA levels by one-way ANOVA. The Tukey post-hoc test was used when appropriate. Data were analysed using JMP7 (SAS Institute Inc., Cary, NC, USA) or SPSS 14.0 (SPSS Inc., Chicago, IL, USA) and significance taken at *p*<0.05.

## Results

### In vitro Study

**Figure 1:**
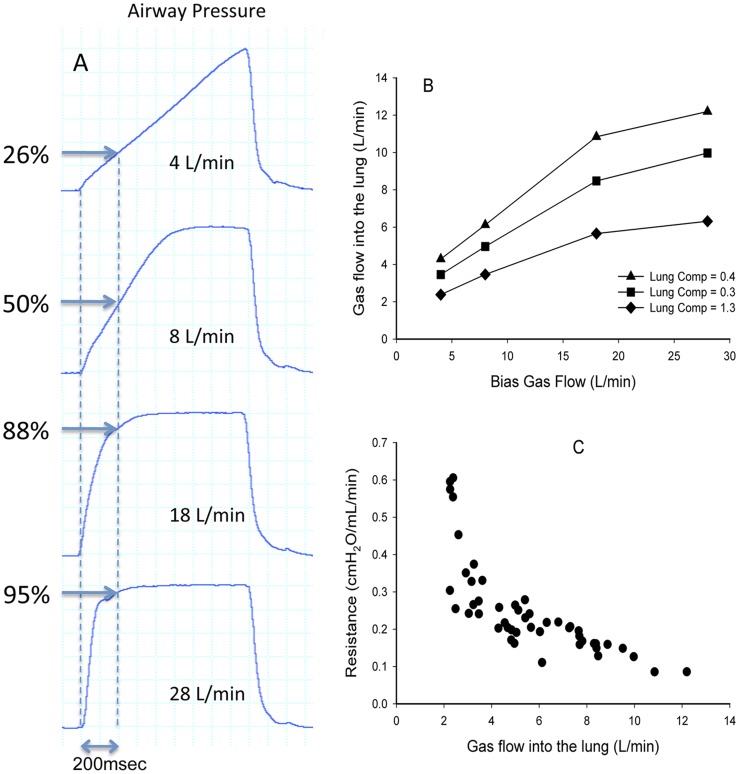
Effects of bias gas flow, lung compliance and airway resistance on pressure and flow in a test lung. (A) Effect of bias gas flow on the pressure wave in the ventilation circuit; dotted line indicates 200 msec after inflation onset and values indicate the pressure at this point, expressed as a percentage of the peak pressure. (B) Effect of lung compliance on gas flow into the lung at different bias gas flows. (C) Relationship between airway resistance and gas flow into the lung, measured at 3 different lung compliances and 4 different bias gas flows.

All three variables (bias gas flow, airway resistance and lung compliance) significantly and independently affected the flow of gas into the test lung. Using the artificial lung with a compliance of 0.8 mL/cmH_2_O and minimal resistance, increasing the bias gas flow from 4 to 8, 18 and 28 L/min increased the average rate of pressure rise from 63.8 cmH_2_O/sec to 93.2, 157.0 and 161.4 cmH_2_O/sec, respectively (Fig. 1A). Thus, 200 msec after the onset of inflation, airway pressure was increased to 26%, 50%, 88% and 95% of the peak inflation pressure (PIP; 35 cmH_2_O), respectively, at the 4 different bias gas flows (Fig. 1A). As a result, airflow into the lungs increased from 3.5 to 5.0, 8.5 and 10.0 L/min, respectively (Fig. 1B) at these different bias gas flows.

Reducing the compliance of the test lung by half (to 0.4 mL/cmH_2_O) reduced airflow into the lung by 30–37% to 2.4, 3.5, 5.7 and 6.3 L/min at ventilator bias flows of 4, 8, 18 and 28 L/min (p<0.0001; Fig. 1B), whereas increasing the compliance to 1.3 mL/cmH_2_O increased the airflow into the lung by 22–27% compared with the lung with a compliance of 0.8 mL/cmH_2_O, to 4.3, 6.1, 10.8 and 12.2 L/min at bias flows of 4, 8, 18 and 28 L/min, respectively (Fig. 1B). Similarly, the relationship between airway resistance and airflow into the lungs was highly significant, following a logarithmic curve (p<0.0001; Fig. 1C).

### Effect of Bias Gas Flow on Cardiorespiratory Parameters

Three animals died from pneumothoraces during the 2-hour ventilation period, two from the 8 L/min group and one from the 18 L/min group. Physiological data are therefore presented for 11 lambs in the 8 L/min group, 11 in the 18 L/min group and 14 in the 28 L/min group.

The peak inspiratory gas flows measured at the wye piece by pneumotachograph in the 8, 18 and 28 L/min bias gas flow groups were 14.9±1.7, 20.8±1.7 and 28.2±1.4 L/min, respectively, confirming that bias gas flow does not accurately reflect gas flow into the lung. As the lambs were ventilated in the PSV+VG mode, in which inspiration ceases when flow reduces to <15% of peak inspiratory flow, the inflation time decreased with increasing bias gas flow (table 1). Peak inspiratory pressure was lowest, whereas tidal volumes and ventilator efficiency index were highest (table 1), in lambs ventilated with a bias gas flow of 8 L/min. Surprisingly, the rate of rise of inspiratory flow was greatest, the expiratory time was shortest and the expiratory flow was greatest in lambs ventilated at 18 L/min (table 1). Pulmonary compliance was similar for all animals (1.8–1.9 mL/cmH_2_O) but pulmonary resistance was highest, and tidal volume lowest in lambs ventilated at 28 L/min (table 1). To maintain pCO_2_ levels during the experiment, the ventilator rate was gradually increased in all animals, independently of bias gas flow (*p*<0.05; data not shown), resulting in a decrease in expiratory time. However, the PaCO_2_ was highest in lambs ventilated at 18 L/min (table 2), despite these lambs also having significantly higher ventilator rates and expiratory airflows (table 1).

**Table 1:** Effect of bias gas flow on ventilatory parameters.

Bias gas flow (L/min)	8 (*n* = 11)	18 (*n* = 11)	28 (*n* = 14)
Ti (s)	0.52±0.01**^‡^	0.41±0.01^‡^	0.38±0.01
Te (s)	0.95±0.03	0.90±0.02^‡^	1.04±0.03
PIP (cmH_2_O)	24.5±0.7**^‡^	29.2±0.7	29.5±0.6
MAP (cmH_2_O)	11.5±0.2	12.7±0.2	12.1±0.2
Rate (breaths/min)	42±1**	47±1^†^	44±0
TV (mL/Kg)	6.91±0.07^†^	6.70±0.07	6.65±0.06
Inspiratory flow (L/min)	14.9±1.7*^‡^	20.8±1.7^‡^	28.2±1.4
Expiratory flow (L/min)	−23.9±1.3**	−30.6±1.2^†^	−26.6±1.1
Δ inspiratory flow (L/s^2^)	1.88±0.2**^†^	3.07±0.18^†^	2.46±0.17
VEI (ml.Torr/kg.min)	0.13±0.01**^‡^	0.08±0.01	0.09±0.01
Resistance (cmH_2_O/L/s)	72.5±3.1^‡^	77.7±2.8	86.0±2.5

Values are mean ± SEM for inflation time (Ti), expiratory time (Te), peak inspiratory pressure (PIP), mean airway pressure (MAP), ventilator rate, tidal volume (TV), inspiratory flow, expiratory flow, rate of rise for inspiratory flow (Δ inspiratory flow), ventilator efficiency index (VEI) and resistance during ventilation at ventilator bias gas flows of 8, 18 or 28 L/min. Data are averaged for all time points. *p<0.05; **p<0.01 vs. flow 18 L/min,^ †^ *p*<0.05 and ^‡^ *p*<0.01 vs. flow 28 L/min.

Heart rate, but not blood pressure, was higher in the 18 L/min group compared with both other groups (table 2). PaO_2_ (table 2), but not mean airway pressure (table 1) or oxygenation index, was also highest in lambs ventilated at 18 L/min, although FiO_2_ (table 2) tended to be higher in these lambs. Pneumothorax rates tended to be higher in lambs ventilated at 18 L/min (66%) compared with lambs ventilated at 28 L/min (43%) and 8 L/min (31%) (table 2). Blood white cell and neutrophil counts were not different between groups, although blood white cell count at 60 and 120 minutes were decreased in all three groups compared with baseline values (*p*<0.01; data not shown).

**Table 2:** Effect of bias gas flow on cardiorespiratory parameters.

Bias gas flow (L/min)	8 (*n* = 11)	18 (*n* = 11)	28 (*n* = 14)
HR (bpm)	151.8±4.6*	184.2±4.1^†^	158.8±3.8
Mean BP (mmHg)	73.7±3.3	66.2±2.9	65.5±2.6
PaO_2_ (mmHg)	54.4±6.6*	82.3±5.5	72.5±4.7
PaCO_2_ (mmHg)	59.3±2.0*^†^	69.9±1.5	65.1±1.3
FiO_2_ (%)	57.8±3.2	66.1±3.0	58.3±2.7
PNX (n/total)	4/13	8/12	6/14

Values are mean ± SEM for heart rate (HR), mean arterial blood pressure (BP), PaO_2_, PaCO_2_, FiO_2_ and pneumothorax (PNX) during ventilation at ventilator bias gas flows of 8, 18 or 28 L/min. Data are averaged for all time points. *p<0.05 vs. flow 18 L/min,^ †^ *p*<0.05 vs. flow 28 L/min.

### Effect of Bias Gas Flow Rate on mRNA Levels of Acute Response Genes and on Pulmonary Histology

**Figure 2:**
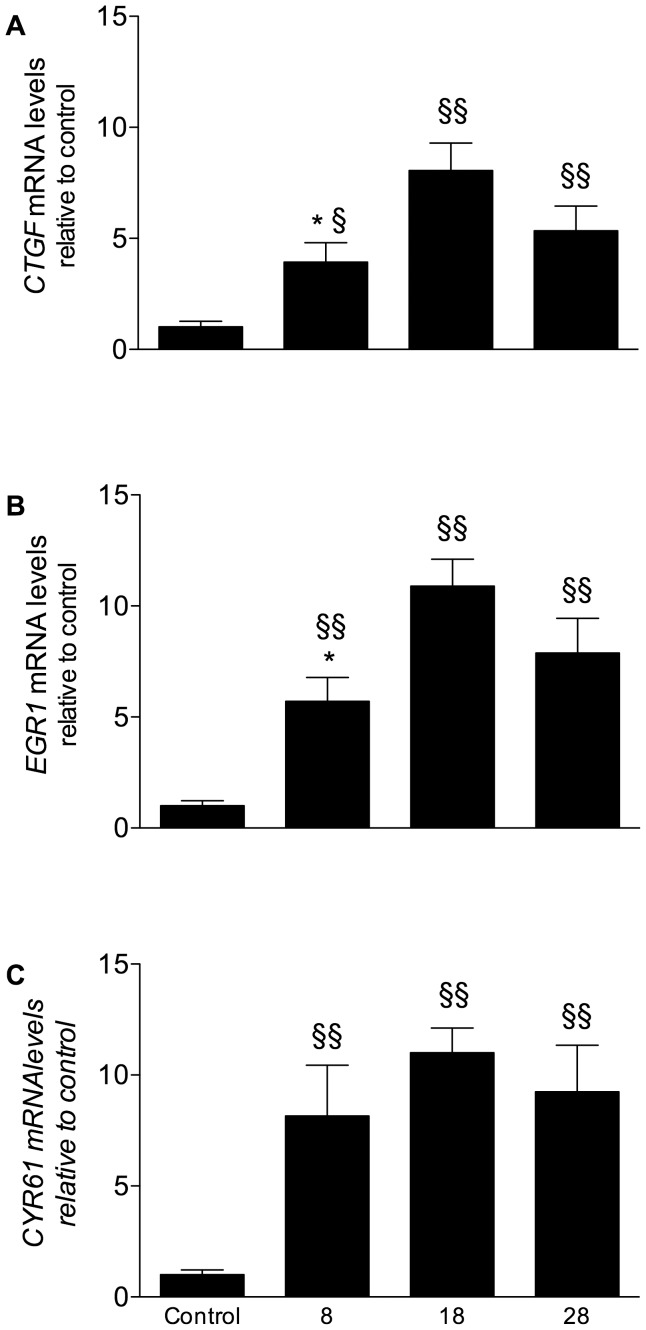
Effect of bias gas flow on mRNA levels of *CTGF*, *EGR1* and *CYR61*. mRNA levels, expressed as fold change relative to mRNA levels in age-matched un-ventilated control tissue (*n* = 8), of *CTGF* (A), *EGR1* (B) and *CYR61* (C) in lung tissue of lambs ventilated with a ventilator bias gas flow of 8 (*n = *11), 18 (*n = *11) or 28 L/min (*n = *14). * *p*<0.05 vs. flow 18 L/min, ^§^ *p*<0.05 vs. control tissue and ^§§^ *p*<0.01 vs. control tissue.

Mechanical ventilation caused significant up-regulation of *CYR61, CTGF* and *EGR1* mRNA levels (control, *n* = 8; 8 L/min, *n* = 11; 18 L/min, *n* = 11; 28 L/min, *n* = 14), but the levels of all 3 genes were highest in lambs ventilated at 18 L/min, consistent with the physiological data. *CTGF* and *EGR1* mRNA levels were ∼200% greater than those seen in lambs ventilated at 8 L/min (*p*<0.05; Fig. 2), whereas mRNA levels of *CYR61* were only 35% higher and this was not statistically significant.

Quantitative histological analysis of the lung (control, *n* = 8; 8 L/min, *n* = 5; 18 L/min, *n* = 5; 28 L/min, *n* = 5) revealed that the percentage of space occupied by tissue was significantly reduced in lambs ventilated at 8 and 28 L/min compared with unventilated control lambs. On the other hand, collagen density, measured as a percentage of tissue area, was increased in lambs ventilated at 8 and 28 L/min compared to control lambs (table 3), which may be related to the reduction in tissue area observed in these lambs.

**Table 3:** Quantitative analysis of the effect of bias gas flow on pulmonary histology.

Bias gas flow (L/min)	Control (*n* = 8)	8 (*n* = 5)	18 (*n* = 5)	28 (*n* = 5)
Elastin (% of lung tissue)	11.4±0.46	10.8±0.49	10.0±0.55^§†^	11.6±0.56
αSMA (% of lung tissue)	17.8±0.77	18.7±1.2*	14.9±0.97^§†^	21.3±1.10^§^
Collagen (% of lung tissue)	0.27±0.01	0.32±0.01^§^*	0.27±0.01^†^	0.32±0.01^§^
Ki67 (% of total cells)	6.46±0.3	5.76±0.40^†^	6.66±0.58	7.52±0.40
tissue ratio (% of total area)	40.0±3.0	34.2±2.9^§^*	41.9±2.8^†^	33.7±3.1^§^

The proportion of lung tissue stained positive for elastin, αSMA and collagen, the proportion of Ki67-positive cells (labelling index, representing mitotic cells) and the proportion of each field of view occupied by tissue rather than air space (using a point counting technique) [7] for age matched non-ventilated controls and animals ventilated at 8, 18 and 28 L/min. ^§^ *p*<0.05 vs. control tissue, * *p*<0.05 vs. flow 18 L/min, ^†^ *p*<0.05 vs. flow 28 L/min.

Ventilation at 18 L/min had the most marked effects on the quantitative histological analysis of lung tissue, with decreased elastin density compared with control lambs and lambs ventilated at 28 L/min, decreased αSMA staining density compared with all other groups and increased proportion of total lung space occupied by tissue compared with lambs ventilated at 8 and 28 L/min (table 3). The proportion of Ki67 positive cells (labelling index) was significantly lower in lambs ventilated at 8 vs 28 L/min (table 3). There was no significant difference amongst groups in number of secondary septal crests (data not shown).

**Figure 3:**
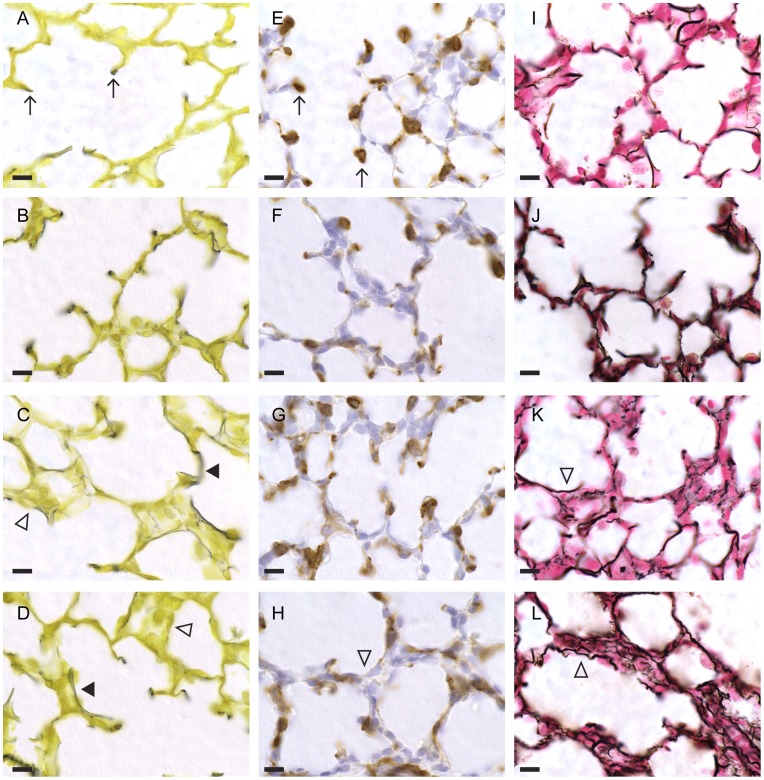
Representative photomicrographs of lung tissue from age matched non-ventilated controls and ventilated animals. Photomicrographs of controls (*n* = 8) are shown in the first row (A, E, I), of lambs ventilated at 8 L/min (*n* = 5) in the second row (B, F, J), 18 L/min (*n* = 5) in the third row (C, G, K) and 28 L/min (*n* = 5) in the fourth row (D, H, L). Columns demonstrate elastin (stained black with Hart’s resorcin stain; A–D), differentiated myofibroblasts (stained brown using immunohistochemistry; E–H), and collagen type I and III fibres (stained black with Gordon-Sweet’s stain; I–L). Bar 10 µm. Arrows (↑) demonstrate secondary septal crests with elastin (A) or myofibroblasts (E) visible at the tip. Solid arrowheads (▴) demonstrate abnormal deposition of elastin and open arrowheads (Δ) demonstrate thickened interstitium (C, D, H) containing a finer meshwork of collagen fibres in tissue ventilated at 18 and 28 L/min (K, L).

Qualitative analysis demonstrated that, in control tissue, elastin and αSMA staining was predominantly located at the tips of the secondary septal crests. Following only 2 h of ventilation, septal crests appeared thicker and shorter and increased elastin and αSMA staining was seen in the alveolar wall with differentiated myofibroblasts more randomly distributed throughout the interstitium (Fig. 3). These changes were most apparent in lambs ventilated at flows of 18 and 28 L/min (Fig. 3). Collagen fibres, seen as thick parallel fibres in the airway wall in control tissue, appeared more tortuous following ventilation with a fine meshwork of fibres in the thickened interstitium. Again, these changes were most apparent in lambs ventilated at 18 and 28 L/min (Fig. 3).

## Discussion

This study has investigated the effects of ventilator bias gas flows on gas flow into the lung, ventilator efficiency, cardiopulmonary responses and lung injury in a preterm lamb paradigm. Although bias gas flow influences the rate of gas flow into the lung, it is only one of a number of determinants of inspiratory flow, with both respiratory system compliance and resistance also having highly significant effects. We report that high bias gas flows, resulting in higher inspiratory gas flows, have adverse effects on ventilatory parameters, increase mRNA levels of early response genes and result in histological evidence of lung injury. These findings occurred after only 2 hours of ventilation and it remains to be determined whether adverse effects would increase with increasing duration of ventilation.

Many factors are known to contribute to the development of VILI in the preterm lung, including barotrauma [8], [9], volutrauma [10], [11], atelectotrauma [9] and oxytrauma [1], [2]. As volutrauma is considered to be more injurious than barotrauma [10], [11], we used volume guarantee ventilation to ensure delivery of a consistent tidal volume independent of changes in compliance or resistance. Compliance was not different amongst groups, although resistance was lowest in lambs ventilated at 8 L/min and highest in lambs ventilated at 28 L/min, most likely secondary to less turbulence with lower flows. Lambs ventilated at 18 and 28 L/min had lower tidal volumes (by 0.3 mL/Kg; less than 5% of the set tidal volume) but required higher PIP values than lambs ventilated at 8 L/min. Apart from flow-related parameters (including the inflation time, which is closely correlated to flow) [17], ventilator rate was the only ventilatory parameter different between lambs ventilated at 18 and 28 L/min; lambs ventilated at 18 L/min were ventilated at higher rates in response to higher PaCO_2_ levels. As there is a pattern of greater lung injury in animals ventilated at 18 L/min compared with lambs ventilated with 28 L/min, despite similar tidal volumes and pressures, it appears that the differences in degree of injury are unrelated to either barotrauma or volutrauma.

A change in the shape of the pressure wave may contribute to lung injury by altering the rate of lung inflation [24]. A slower pressure rise can be achieved by altering various ventilatory parameters [25], [26], [27], including reducing the bias gas flow, which reduces gas flow into the lung by reducing the pressure gradient between the ventilator circuit and the lung throughout most of inflation (Fig. 1A). However, it should also be noted that ET tube resistance and even respiratory system compliance influences the rate of pressure increase, presumably due to their independent effects on gas flow into the lungs (Fig. 1B & 1C). A decreased bias gas flow, with its attendant altered pressure wave pattern, has been shown to protect against lung injury in adult sheep ventilated at high pressures (PIP = 50 cmH_2_) [28]. However, there are no studies in neonates reporting advantages of one pressure wave pattern over another.

Lambs ventilated at 18 L/min had the most marked injury, despite having lower inspiratory flows than lambs ventilated at 28 L/min. Possible reasons for this include a contribution from hyperoxia-induced trauma [1], [2], as the FiO_2_ and PaO_2_ were highest at 18 L/min, and higher ventilatory rates secondary to higher PaCO_2_ values, a finding which may also have contributed to the trend in a higher rate of pneumothoraces in these lambs.

Inflation time, which has been associated with risk of pneumothorax in preterm babies [25], [29], was inversely related to bias gas flows in this study.

However, the rates of increase in both inspiratory and expiratory flows were highest in lambs ventilated with 18 L/min bias gas flow, rather than in those ventilated with 28 L/min. The finding of most marked lung injury and highest mRNA levels of the acute response genes in lambs ventilated with 18 L/min, therefore, supports the hypothesis that lung injury may be related to rate of rise of flow and/or expiratory flow.

The lower rate of rise of inspiratory flow in lambs ventilated at 28 L/min compared with those ventilated at 18 L/min is most likely due to turbulent gas flow in the endotracheal tube at a flow of 28 L/min. Flow of gas at 37°C and at a barometric pressure of 760 mmHg becomes turbulent through a size 4.0 endotracheal tubes once the flow exceeds 18 L/min [30]. Thus, at the highest flow of 28 L/min the flow pattern will have been turbulent, paradoxically leading to a lower rate of increase in flow into the airways. Furthermore, a turbulent flow pattern leads to a larger pressure drop from the wye piece to the endotracheal tube [30]. Thus, even though PIP values measured by the sensor at the wye piece were similar in lambs ventilated with flows of 18 and 28 L/min, the pressure in the endotracheal tube is likely to have been higher at 18 L/min when flow is more laminar than at 28 L/min. Lambs ventilated at 18 L/min also had the greatest expiratory flow. The reason for this is not clear, but may result from higher expiratory flow in the endotracheal tube creating a Venturi-like effect at the intersection between the comparatively narrow tube and wider ventilator circuit. It could also be due to reduced pulmonary compliance as a result of greater lung injury. We did not measure dynamic pulmonary compliance, but there was no difference in effective pulmonary compliance between lambs ventilated at 18 and 28 L/min. However, it is unlikely that the system is sensitive enough to detect the changes in compliance that could lead to these changes in flow.

Heart rate was significantly higher in animals ventilated at 18 L/min. As tidal volume in animals ventilated at 18 L/min was not different from animals ventilated at 28 L/min, this is unlikely to be hyperexpansion leading to altered diastolic filling of the heart. It may reflect higher PaCO_2_ values in this group which, despite increasing the ventilator rate, remained above 60 mmHg. Since the animals were muscle relaxed, they were not able to increase their breathing rate spontaneously to compensate for higher PaCO_2_, but the heart rate may have increased via the chemoreceptor reflex in an attempt to eliminate surplus arterial CO_2_ [31].

The early response genes *CTGF*, *CYR61* and *EGR1* have recently been identified as potentially useful biomarkers of VILI in preterm newborn lambs [21]. They are also up-regulated in lungs of adults with chronic obstructive pulmonary disease and pulmonary fibrosis [18], [19], [20], in response to stimuli promoting fetal lung growth in sheep [32] and in response to hyperoxia in mice [33]. CTGF and CYR61, members of the CCN family of cysteine-rich proteins, promote cell proliferation and differentiation, tissue regeneration and synthesis of extra-cellular components, including collagen, and are involved in embryonic angiogenesis [34], [35]. CTGF is immunolocalised to activated fibroblasts and type II alveolar epithelial cells [20] whereas CYR61 is an extracellular matrix associated protein [36], [37] and is up-regulated when subjected to cyclic mechanical stretch [36]. CYR61 mediates expression of αSMA in smooth muscle cells through cytoskeletally based mechano-transduction [36].

Expression of the transcription factor EGR1 is rapid and of short duration after acute tissue injury [18], hypoxia [38] and pneumonectomy [39], although a prolonged expression has been found in lung tissue from patients suffering from advanced stage emphysema [18]. Thus, EGR1 is involved in both immediate early responses and more chronic lung destruction.

We report an up-regulation of mRNA levels of these three genes in the lung after 2 hours of ventilation, which is consistent with our previous study demonstrating that levels are elevated within 15 minutes and remain elevated after 2 hours of injurious ventilation in preterm lambs [21]. In our study, highest levels (∼10 fold of control values) were measured in animals ventilated at 18 L/min. In both studies, up-regulation of these genes occurred shortly after the onset of ventilation and higher expression levels were associated with ventilation using larger volumes or higher bias gas flows. Therefore, altered levels of these early response genes likely reflect the level of injury in the preterm lung and might potentially predict the level of injury and risk of BPD development in preterm infants.

The histological evidence of lung injury that we report is consistent with the gene expression data. Secondary septal crest formation is an important part of normal lung development, during which the distal airsacs subdivide to form alveoli. An arrest of alveolarisation is a primary characteristic of BPD [3], [4] and is associated with abnormal deposition of elastin [3], [4], [5] and collagen fibres [4], [6], decreased secondary septal crest densities [7] and increased mesenchymal cell proliferation [4], [7].

Collagen and elastin fibres provide the stabilising framework for the lung [40] and both are important for secondary septal crest formation and airway branching [41], [42]. Elastin is essential for lung recoil [43] and is deposited primarily at the tip of secondary septal crests forming an elastin ring around the alveolar opening. Abnormal elastin deposition (increased deposition and altered spatial pattern) is a well described feature of VILI leading to BPD-like changes in lung tissue architecture [3], [5], [44]. Allison *et al* suggested that VILI mechanically disrupts immature secondary septal crests, causing them to flatten, with the consequence that pre-existing elastin fibres located at the tip of secondary crests are re-absorbed into the primary alveolar wall giving the appearance of abnormal deposition [7].

We describe altered deposition of both elastin and collagen following ventilation, which was most marked at higher flows. Quantitative analysis demonstrated that elastin and collagen densities were lowest after ventilation at 18 L/min, which may reflect disruption of fibres and re-absorption into the primary alveolar wall. Alternatively, it is possible that the altered deposition, with less discrete areas of staining, resulted in an underestimation of the degree of staining by the image analysis software.

The pattern of differentiated myofibroblasts, detected by αSMA staining, followed that of elastin. That is, most myofibroblasts were located at the tip of secondary septal crests in control animals but were more randomly distributed throughout the interstitium following ventilation, consistent with the hypothesis of re-absorption of septal crests into the primary wall [7]. However, we did not find decreased secondary septal crest density in this study, possibly because of the short duration of the study and the possibility that a greater proportion of the septal crests are more mature and robust compared with the very immature lungs described previously [16]. The appearance of shorter and thicker septa after ventilation, especially at the higher flows, might reflect early lung injury and inflammation, as described in baboons with BPD [4]. An actual decrease in density in our study might have been found after a longer duration of ventilation.

Increased cellular proliferation is another characteristic feature of BPD, and has been described in baboons with BPD [4] and following *in-utero* ventilation of 110 day lamb fetuses [7]. Recruitment of inflammatory cells to the lungs, with a consequent fall in circulating neutrophil count [45], has also been reported with ventilation resulting in an increased DNA concentration in the lung [7]. Our findings of increased numbers of proliferating cells, increased space occupied by tissue in the lung and reduced circulating white blood cells are consistent with these reports.

The histological changes that we describe here after only two hours of ventilation are consistent with changes found in babies with BPD [6], [46], [47] and animals with VILI [3], [4], [7]. Our results indicate that lower bias gas flows, even for a short duration of ventilation, may potentially reduce lung injury in preterm infants. We previously demonstrated flows as low as 5 L/min to be safe and efficient in healthy term lambs. Furthermore, optimal ventilator efficiency occurred at flows between 1.5–3 L/Kg.min (bodyweight ∼ 6 Kg) [17]. Lambs of our breed are ∼ 4.5–5 Kg at 131 days’ gestation; therefore, we chose 8 L/min to be the lowest flow in this study. The Dräger Babylog 8000*plus* ventilator can provide continuous bias gas flows from 2–30 L/min. However, at flows of 30 L/min, the flow sensor measurements are inconsistent and we therefore used a maximum flow of 28 L/min. It remains to be determined if further reducing the bias gas flow will result in a further beneficial effect on lung injury whilst still maintaining effective ventilation. Randomised studies in preterm babies will be necessary to determine whether bias gas flows contribute to lung injury in humans.

### Conclusions

High ventilator bias gas flows contribute to more severe lung injury, alter ventilator parameters and up-regulate biomarkers of VILI in the preterm lamb compared to lower gas flows. Paradoxically, lung injury at the highest bias gas flow (28 L/min) was less than at 18 L/min, probably reflecting turbulent gas flow at the highest flows which may mitigate some of the adverse effects of fast laminar flow. These data demonstrate that ventilator bias gas flow is important in the development of VILI. More research needs to be done to examine the effect that rheotrauma has on the more immature lung and to determine whether bias gas flows may contribute to long-term lung injury, such as BPD.
